# Inflammation mechanism and research progress of COPD

**DOI:** 10.3389/fimmu.2024.1404615

**Published:** 2024-08-09

**Authors:** Jiao Xu, Qingyue Zeng, Shuangqing Li, Qiaoli Su, Hong Fan

**Affiliations:** ^1^ General Practice Medical Center, West China Hospital, Sichuan University, Chengdu, China; ^2^ Department of Respiratory and Critical Care Medicine, West China Hospital, Sichuan University, Chengdu, China

**Keywords:** chronic obstructive pulmonary disease, chronic inflammation, inflammatory cells, oxidative stress, systemic inflammation

## Abstract

Chronic obstructive pulmonary disease (COPD) is a common respiratory disease characterized by irreversible progressive airflow limitation, often manifested by persistent cough, sputum production and other respiratory symptoms that pose a serious threat to human health and affect the quality of life of patients. The disease is associated with chronic inflammation, which is associated with the onset and progression of COPD, but anti-inflammatory therapy is not first-line treatment. Inflammation has multiple manifestations and phenotypes, and this heterogeneity reveals different patterns of inflammation, making treatment difficult. This paper aims to explore the direction of more effective anti-inflammatory treatment by analyzing the nature of inflammation and the molecular mechanism of disease occurrence and development in COPD patients, and to provide new ideas for the treatment of COPD patients.

## 1 Introduction

Chronic obstructive pulmonary disease (COPD) is the third cause of death in the world, characterized by high morbidity and mortality, and has become a major disease burden worldwide (1, 2). Chronic inflammation plays an important role in the occurrence and development of COPD, mainly affecting the lung parenchyma and surrounding airway, leading to persistent respiratory symptoms and irreversible progressive airflow restriction (3). This chronic inflammation is characterized by an increase in the number of cells, including macrophages, lymphocytes, and neutrophils, which are mainly located in the pulmonary blood vessels, peripheral airways, and lung parenchyma. In some patients, there may also be an increase in the number of cells, such as eosinophils, T helper 2 (Th2) cells, or type 2 intrinsic lymphocytes (ILC2). These cells can release different inflammatory mediators together with other structural cells (4). Oxidative stress plays a key role in COPD related inflammation, as well as in smokers. COPD patients may also experience systemic inflammation, which exacerbates the severity of cardiovascular, endocrine and metabolic complications. Accelerated lung aging in COPD patients also causes senescent cells in the lung to release inflammatory proteins, including tumor necrosis factor alpha (TNF-α), Interleukin (IL)-1, IL-6, chemokine (C-X-C motif) ligand 8 (CXCL8), chemokine (C-C motif) ligand 2 (CCL2) and matrix metalloproteinases (MMPs). In the progress of diagnosis and treatment of diseases, it is very important to identify the disease phenotype with the best curative effect, and it is also important to explore biomarkers to identify this disease phenotype (4).

## 2 Pathology of COPD

**Figure 1:**
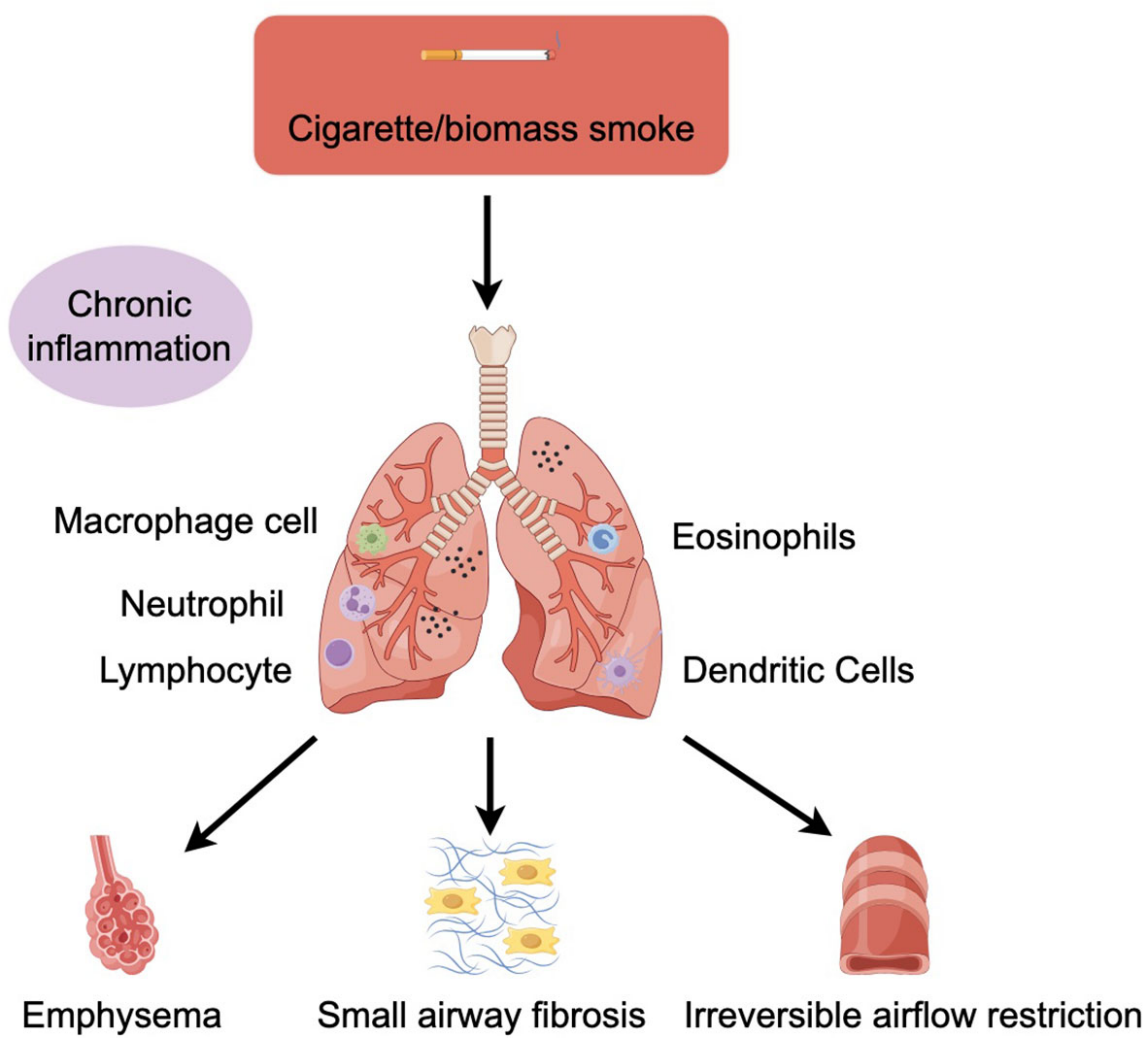
COPD is mainly associated with cigarette/biomass smoke. Cigarette/biomass smoke can cause inflammation of the lung parenchyma and airways, activating macrophages, neutrophil, lymphocyte, eosinophils, and dendritic cells in response to toxic particles in the smoke. Various inflammatory factors can lead to tissue destruction and then to emphysema, impairing the defence and repair function of the airways and ultimately leading to small airway fibrosis and progressive irreversible airflow limitation.

The two main pathological types of COPD are small airway disease due to fibrosis around the bronchioles and emphysema due to destruction of the alveolar walls, which can be distinguished by computed tomography (CT) examination. Although some patients mainly present with small airway disease or emphysema, most patients have a mixed condition (5). These pathological changes are mainly caused by chronic inflammation. Inhalation of harmful gases or particles can cause inflammation of lung parenchyma and respiratory tract. Various inflammatory factors can lead to tissue destruction and then emphysema, damage the defense and repair function of respiratory tract, and eventually lead to small airway fibrosis and progressive irreversible airflow obstruction (6) (**Figure 1**). Usually, the more severe COPD symptoms are, the inflammation of the airway is also correspondingly increased and continues after smoking cessation (1). Even in mild patients, peripheral airway obstruction and loss can occur (7). The emphysema observed in smokers first appears close to the thickened and narrowed bronchioles that are the main obstructions in COPD, and the mechanism by which the small airways thicken so close to the lung tissue destroyed by emphysema remains unclear (6).

## 3 Characteristics of inflammation associated with COPD

Patients with COPD often have a characteristic inflammatory pattern, with a significant increase in the number of macrophages, neutrophils, T lymphocytes and B lymphocytes in airway secretions (8–10). This pattern of inflammation involves both innate and acquired immune responses (i.e. cellular and humoral immunity), which are linked by passive activation of dendritic cells. Barnes et al. (4) showed that COPD patients had a more severe inflammatory response than smokers without airway obstruction, and this inflammatory pattern, once established, persisted even after smoking cessation. Although smoking is a major environmental risk factor for COPD, only a proportion of smokers develop the disease (11). This may be due to differences in the response to smoking among individuals, including factors such as genetic susceptibility, epigenetic changes, and oxidative stress (10). These factors may amplify the inflammation caused by smoking. Studies have shown that inhalation of harmful gases or particles can stimulate macrophages and airway epithelial cells to release a variety of chemokines, and monocytes, neutrophils and lymphocytes can accumulate in the lungs under the influence of a variety of chemokines. This inflammation is still present in people who have stopped smoking. So far, the mechanism of this phenomenon is not clear (12). It has been suggested that this persistent inflammation may be related to abnormalities in the regulation of the immune system and individual differences (4).

### 3.1 Inflammatory cells

The formation of lung inflammation in patients with COPD is mainly associated with congenital and adaptive immunity, among them, the congenital immune contains neutrophils and macrophages, eosinophils and mast cells, natural killer cells, γδ T cells, such as inflammation, fine dominant immune response, adaptive immunity refers to T and B lymphocytes or dominant immune response. In this group of patients, the number of macrophages, neutrophils, and lymphocytes is increased, and these cells are mainly located in the pulmonary blood vessels, peripheral airway, and parenchyma of the lung. However, some patients may also show an increase in the number of eosinophils, ILC2, or Th2 cells. At the same time, airway and alveolar epithelial cells and endothelial cells and other structural cells are also activated. Inflammatory cells can release different inflammatory mediators together with other cells (such as epithelial cells and other structural cells) (4). Studies have confirmed that the lack of local IgA can indirectly lead to bacterial translocation and small airway inflammation, which develops into airway remodeling and eventually irreversible airflow restriction (13).

### 3.2 Macrophages

**Figure 2:**
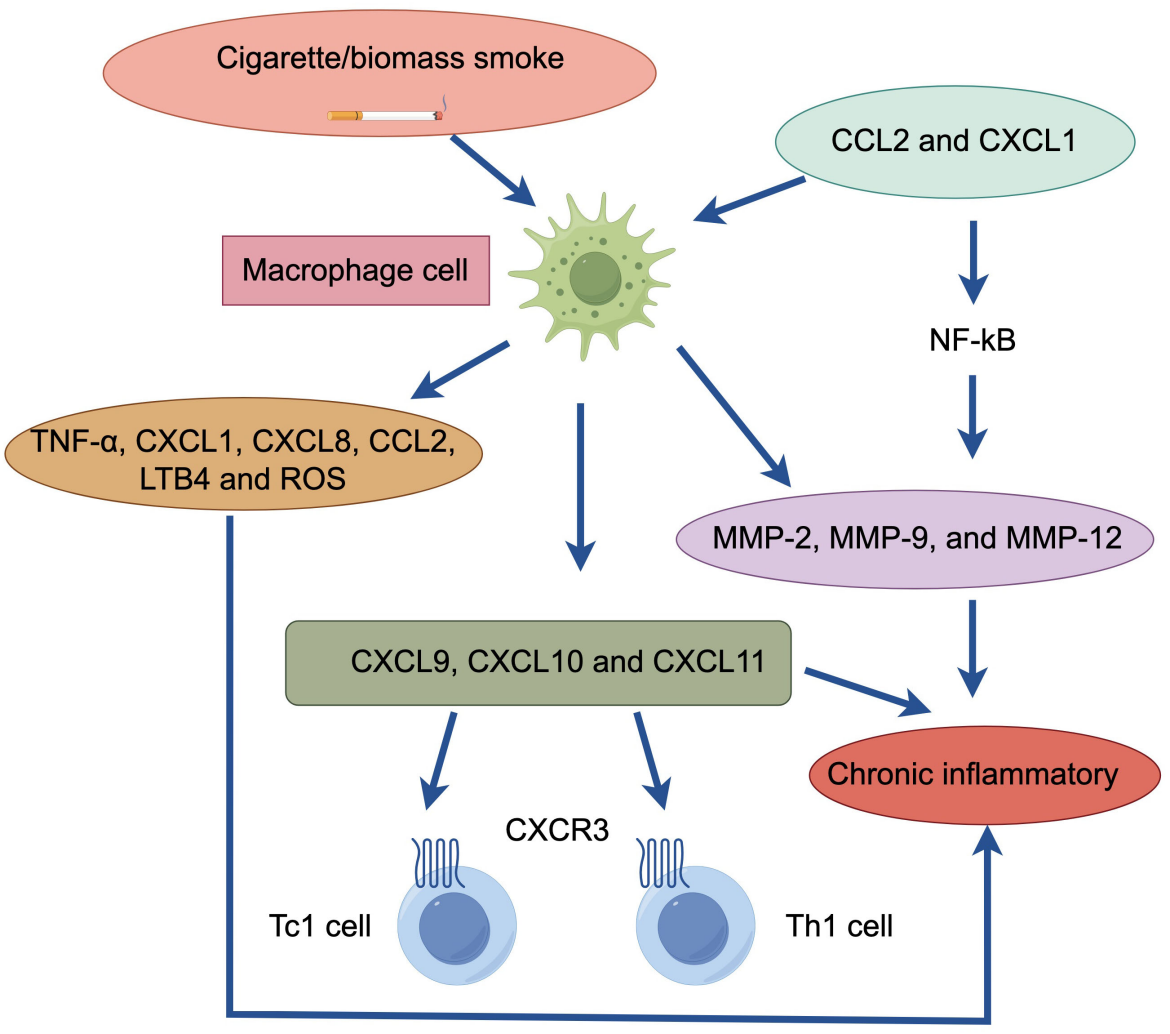
Macrophage cell in patients with COPD. Cigarette/biomass smoke activate macrophages. These macrophages release inflammatory mediators (TNF-α, CXCL1, CXCL8, CCL2, LTB4, ROS) and produce elastolytic enzymes (MMP-2, MMP-9, MMP-12), regulated by the transcription factor NF-κB. The chemokines (CCL2, CXCL1) produced by macrophages enhance the chemotactic response of monocytes, which respond strongly to CXCL1, further attracting macrophages. Additionally, macrophages generate chemotactic effects on CD8+ Tc1 and CD4+ Th1 cells (via CXCL9, CXCL10, CXCL11). This cascade of events ultimately leads to chronic inflammation.

Macrophages play an important role in the chronic inflammation of COPD patients (**Figure 2**). The number of macrophages in the bronchoalveolar lavage fluid (BALF) and sputum of COPD patients can be significantly increased, typically reaching 5-10 times higher than normal values. Macrophages often accumulate at sites of alveolar wall destruction, a phenomenon we can observe in patients with emphysema and COPD, where the number of macrophages correlates with the severity of emphysema. Studies of this phenomenon have shown that macrophages are activated by factors such as cigarette smoke extracts and release inflammatory mediators such as TNF-α, CXCL1, CXCL8, CCL2, leukotriene (LT) B4 and reactive oxygen species (ROS), etc., which mediate the inflammatory response (14). Large increases in macrophages are seen in COPD patients and smokers and are mainly associated with increased circulating chemokines CCL2 and CXCL1, both of which are also found to be significantly elevated in the sputum and BALF of COPD patients (15).

Macrophages have different phenotypes with different activation pathways that lead to different organismal responses. Previous studies have shown that mouse M1-type macrophages are pro-inflammatory, whereas M2-type macrophages are anti-inflammatory, release IL-10, and exhibit significant phagocytic activity (16). However, these differences were not evident in human macrophages, probably because the macrophages in COPD patients are mainly M1-type, but further studies are needed to prove this (17).

Macrophages can secrete elastolytic enzymes, including MMP-2, MMP-9, and MMP-12 (18). MMP-9 is produced by alveolar macrophages and is one of the molecules that mediates the inflammatory response in COPD patients. Compared to smokers, alveolar macrophages in COPD patients secrete more inflammatory proteins and have higher elastolytic activity, suggesting increased activation of macrophages in COPD patients (19). Much of the upregulation of inflammatory proteins is regulated by the transcription factor NF-κB in macrophages, which is extensively activated when the disease is exacerbated, resulting in massive production of inflammatory proteins (20). This inflammatory protein can also be further secreted upon exposure to cigarette smoke, thereby accelerating the rate of inflammation. Macrophages mediate this mechanism even when kept continuously in culture for more than 3 days, so that macrophages in smokers and nonsmokers are fundamentally different (4).

Monocytes from COPD patients exhibit a stronger chemotactic response to CXCL1 than monocytes from smokers and non-smokers (21). In addition, macrophages can also exert chemotactic effects on CD8+ Tc1 and CD4+ Th1 cells by binding to CXCR3, a chemokine receptor expressed by CD8+ Tc1 and CD4+ Th1 cells, to release CXCL9, CXCL10 and CXCL11 chemokines (22).

It has been shown that in healthy individuals and smokers, corticosteroids inhibit the release of CXCL8, TNF-α and MMP-9 from macrophages, thereby alleviating the inflammatory response, but in patients with COPD, where the inflammatory response is mainly mediated by cytokines, chemokines and proteases, corticosteroid use is ineffective (23) and fails to work in macrophages of COPD patients, showing a relatively ineffective (19). Resistance to corticosteroids in COPD patients may be due to decreased histone deacetylase (HDAC) 2 activity due to increased secretion of cytokines (e.g., TNF-α and CXCL8) in macrophages (24).

Both alveolar-derived macrophages and monocyte-derived macrophages in patients with COPD exhibit reduced phagocytic uptake of bacteria, which may be the result of long-term colonization of the lower airways by bacteria such as Haemophilus influenzae or Streptococcus pneumoniae (25), which is present in at least 50% of patients with COPD and may increase the risk of acute exacerbation of COPD, in addition to causing chronic inflammation (26). In addition, macrophage phagocytosis, such as phagocytosis of apoptotic cells, is also defective in COPD patients, which may contribute to the persistence of inflammation in COPD patients (27). The cause of the defective phagocytosis of macrophages is still unclear, and some findings suggest that it may be related to the defective function of cellular microtubules, which are closely related to cytophagy (28).

### 3.3 Neutrophils

Increased sputum and BALF neutrophilia is a typical feature of COPD, and this neutrophil-induced inflammation can caused by regular exposure to cigarette smoke, infectious agent and oxidative stress (5). Neutrophil recruitment to the airways and parenchyma involves initial adhesion to endothelial cells through E-selectin, which is upregulated on endothelial cells in the airways of patients with COPD. Adherent neutrophils migrate into the respiratory tract under the direction of various neutrophil chemotactic factors, including LTB4, CXCL1, CXCL5, and CXCL8, levels of which are increased in airways of patients with COPD (4).

Neutrophils secrete several serine proteases including neutrophil elastase (NE), cathepsin G, proteinase-3, MMPs and myeloperoxidase (MPO) which may contribute to the alveolar damage (4, 29). Alpha‐1 antitrypsin (AAT) is a serum inhibitor of serine proteinases. AAT deficiency (AATD), which is due to a genetic abnormality in the antiproteases, is associated with neutrophilic inflammation in the lung and accounts for less than 1% of COPD patients (5). AATD that results in early onset emphysema, usually in smokers, due to inactivate NE and other serine proteases (30). Despite these theoretical mechanisms, however, only a few patients have AATD, and, thus, it has been difficult to explain why the lung is not normally protected in the majority of COPD patients.

It has been suggested that the size and extent of the neutrophil traffic may be related to the pathological changes that occur in individuals with COPD in case of emphysema (31). The NE is stored within neutrophil azurophil granules which diffuse away from the granule until the concentration has fallen sufficiently for the enzyme to be completely inactivated by the local concentration of AAT. AAT limits rather than prevents neutrophil protease-related damage. It binds to proteases via a one-to-one molar ratio. However, at the location where a degranulating neutrophil is located, the concentration of NE is much higher than that of AAT. This results in obligatory tissue damage around each degranulating cell until the concentration of protease is reduced by diffusion into the local tissue environment. Thus, the role of AAT is to slow down rather than completely stop the damage caused by these proteases (32). This theoretical relationship may explain not only the tissue destruction that occurs in the absence of AATD, but also the extensive destruction seen in deficient subjects. It can be predicted that in people who do not have AATD, there will always be some tissue damage in the immediate vicinity of a degranulating neutrophil. When the AAT concentrations are significantly reduced (as in patients with AATD), the area of damage that will occur can be predicted to be far greater. This would explain not only the increased susceptibility of patients with AATD to tissue damage, but it also provides an explanation for the development of similar but limited damage in non-AATD subjects.

**Figure 3:**
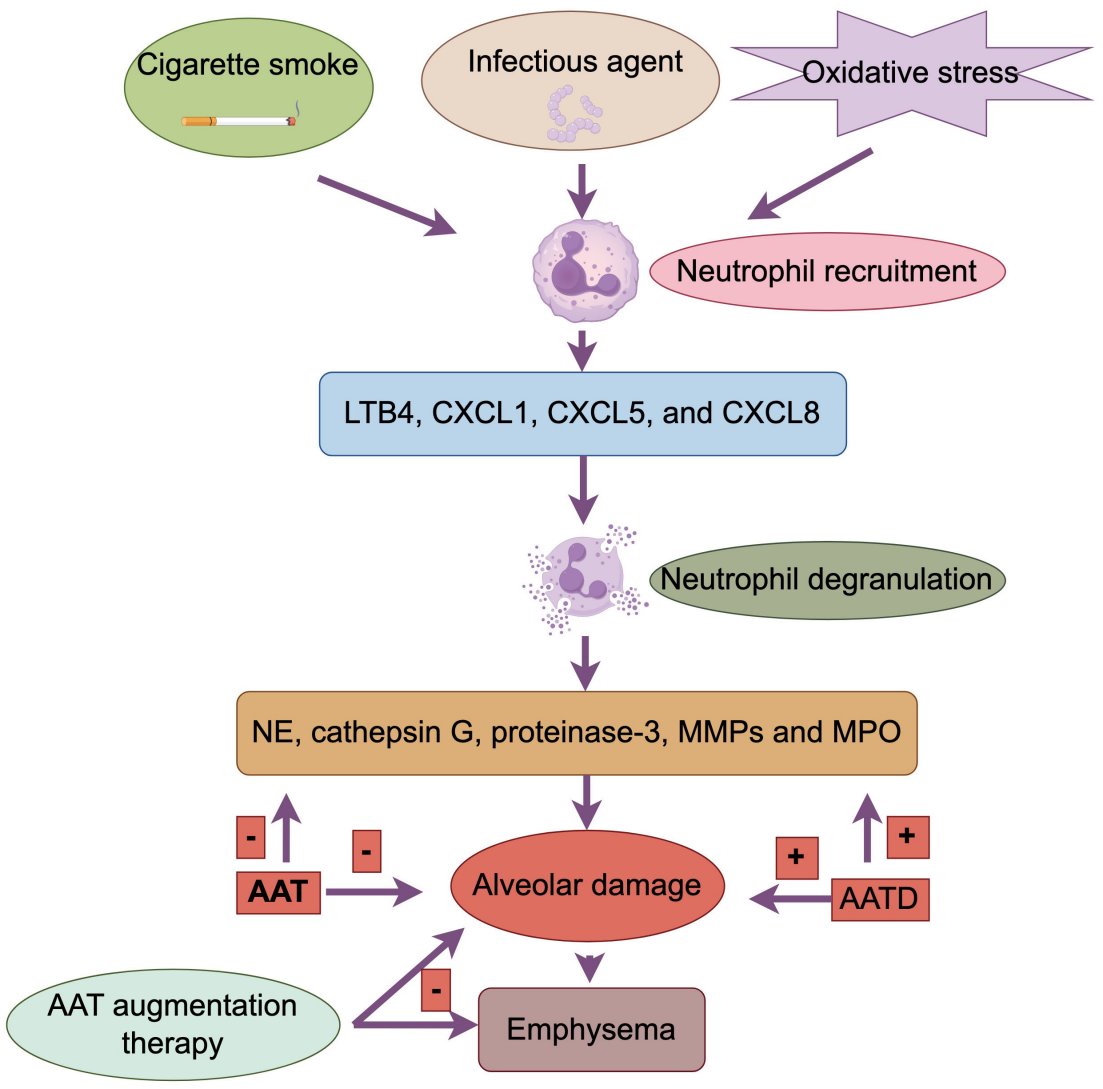
Neutrophil in patients with COPD. Cigarette smoke, infectious agents, and oxidative stress induce neutrophilia. Neutrophils migrate to the respiratory tract under the guidance of neutrophil chemotactic factors (LTB4, CXCL1, CXCL5, CXCL8). Neutrophils secrete serine proteases (NE, cathepsin G, proteinase-3, MMPs, MPO), leading to alveolar damage. Alpha-1 antitrypsin (AAT) inhibits serine proteases. AAT deficiency (AATD) results in insufficient inactivation of NE and other proteases, causing greater tissue damage. AAT, by binding to proteases, slows but does not completely prevent tissue damage. AAT augmentation therapy can slow the progression of COPD.

The above findings have implications for the precise treatment of COPD. The World Health Organization (WHO) recommends that especially in areas with a high prevalence of AATD, all patients with COPD should be screened for AATD (1). AAT augmentation therapy with infusion of human plasma derived purified AAT has been shown to slow progression (33) (**Figure 3**).

### 3.4 Eosinophils

As a heterogeneous lung lesion, COPD has multiple subtypes. With the deeper understanding of the inflammatory mechanisms of COPD, it has been found that neutrophilic inflammation is the most common type of inflammation, but 20% to 40% of patients also belong to the type 2 inflammatory endotype, characterized by elevated eosinophils (EOS) (34, 35). It is generally accepted that type 2 inflammation is characterized by upregulation of the expression of type 2 cytokines, such as IL-4, IL-5, and IL-13, produced by Th2 and ILC2 (36). Currently, the mechanism of airway type 2 inflammation formation in COPD is unclear. Some studies suggest that granulocyte-macrophage colony-stimulating factor (GM-CSF) and CCL5 secreted by the airway epithelium in patients with COPD may promote the survival and recruitment of EOS in the airways (37). Acute exacerbations are more frequent in patients with COPD characterized by type 2 inflammation compared with the predominantly neutrophil-driven, non-type 2 inflammatory endotype (35).

The differences in eosinophil counts may determine different responses to inhaled corticosteroid (ICS) therapy (38). By modeling eosinophil count as a continuous variable, a *post hoc* analysis showed that ICS-containing regimens had little to no effect when blood eosinophil count was less than 100 cells/μL, and thus this threshold could be used to identify patients with a low likelihood of benefit from treatment with ICS (39). In addition, decreased eosinophils in blood and sputum were associated with an increase in proteobacteria, primarily Haemophilus, as well as an increase in bacterial infections and pneumonia (40, 41). ICS are anti-inflammatory drugs used in combination with one or two long-acting bronchodilators (LABDs) for the treatment of COPD. Studies have shown that ICS reduce exacerbation rates, improve quality of life, and prevent mortality in COPD patients with a history of exacerbations (42, 43). Accordingly, GOLD recommends the use of eosinophil testing for COPD patients with a history of exacerbations in clinical practice, despite their appropriate use of LABDs, to identify patients who are best suited to receive ICS therapy (1). The threshold of a blood eosinophil count ≥ 300 cells/μL identifies the top of the continuous relationship between eosinophils and ICS, and can be used to identify patients with the greatest likelihood of treatment benefit with ICS. The thresholds of < 100 cells/μL and ≥ 300 cells/μL should be regarded as estimates, rather than precise cut-off values, that can predict different probabilities of treatment benefit (38, 44). There is no clear evidence that ICS treatment reduces blood eosinophil count, so it retains their predictive value independent of ICS treatment.

**Figure 4:**
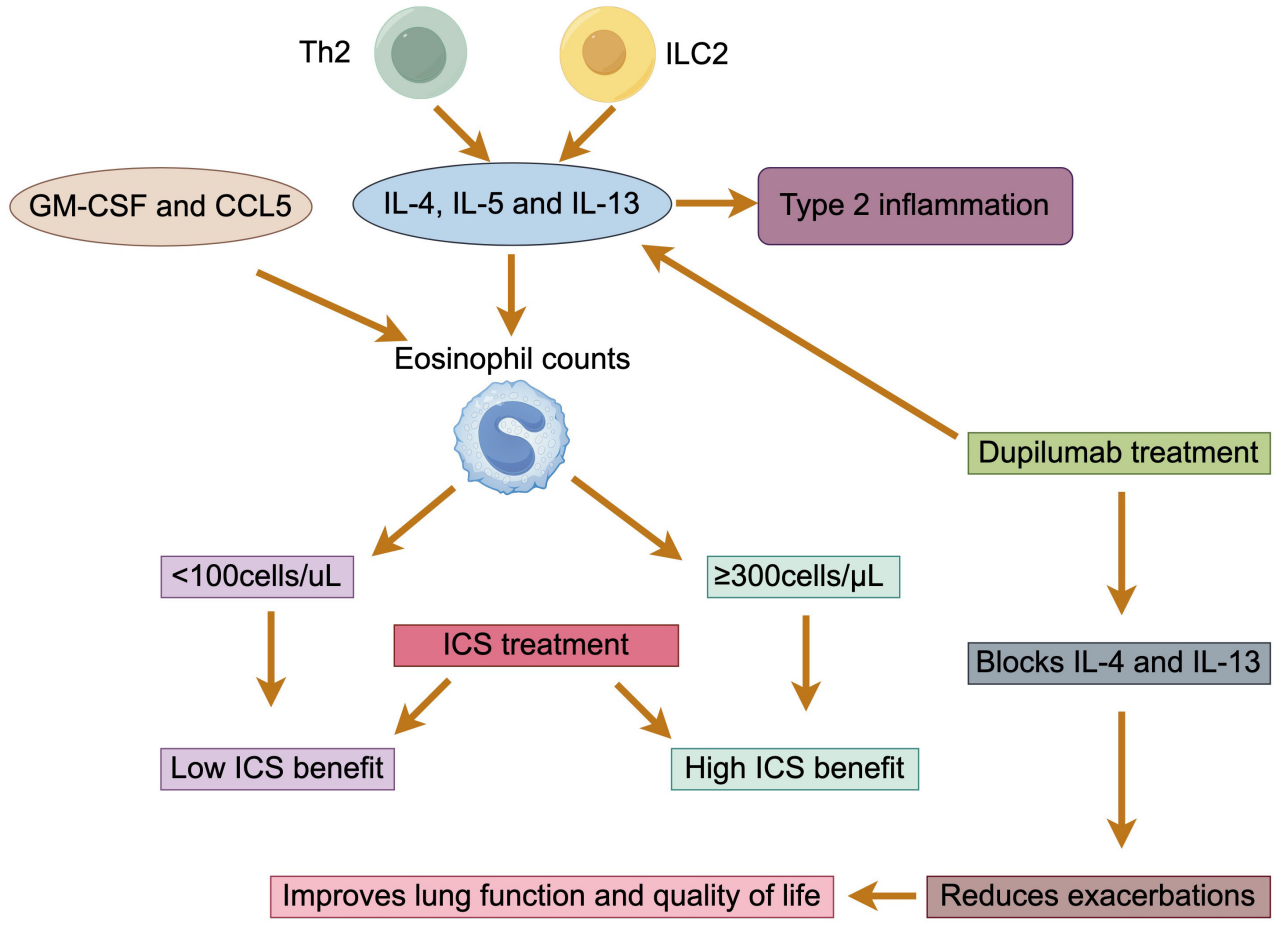
Eosinophils in patients with COPD. The type 2 inflammatory endotype is characterized by the production of IL-4, IL-5, and IL-13 by Th2 and ILC2 cells. GM-CSF and CCL5 facilitate the survival and recruitment of eosinophils. The effectiveness of ICS therapy is correlated with eosinophil counts: less than 100 cells/μL indicates low effectiveness, while counts of 300 cells/μL or higher indicate high effectiveness. Dupilumab, which blocks IL-4 and IL-13, can reduce exacerbations and improve both lung function and quality of life.

The pathogenesis of patients with COPD is complex, and although it is currently recommended that group E patients with blood eosinophil count ≥300/μL should be treated with the triple therapy of “ICS + LAMA + LABA”, some patients are still poorly controlled after the triple therapy [1]. The New England Journal recently published a new clinical study revealing the efficacy of using dupilumab in the treatment of patients with COPD with type 2 inflammatory features. Dupilumab, a fully human monoclonal antibody, blocks the shared receptor component for IL-4 and IL-13, key drivers of type 2 inflammation. The results showed that in COPD patients with type 2 inflammation as indicated by elevated blood eosinophil counts, those who received dupilumab had fewer exacerbations, better lung function and quality of life, and less severe respiratory symptoms than those who received placebo (45) (**Figure 4**). In summary, biologically targeted therapy for type 2 inflammation may be a new treatment option.

### 3.5 Lymphocytes

#### 3.5.1 T lymphocytes

Lymphocytes play an important role in the mechanism of COPD. Smoking status, degree of airflow obstruction and emphysema were all associated with increased CD8+ cell counts and/or CD8+/CD4+ ratios (46). Numerous studies have found increased numbers of CD8+ T-lymphocytes in the blood and lower respiratory tract tissues of patients with COPD compared to controls, as well as in sputum and BAL, but the number of lymphocytes in these secretions is insignificant and very difficult to count (46, 47). CD4+ T cells differentiate into different functional T cell subsets upon activation, including Th1, Th2, Th9, Th17, Th22, Tfh, and regulatory T (Treg) cells, and CD8+ T cells also differentiate into diverse subsets, including Tc1, Tc2, Tc9, Tc17, Tc22 cells, Tfcs, and suppressive CD8 Tregs (48), of which Th1/Tc1, Th2/Tc2, Th17, Treg are closely associated with COPD (49).

Th1/Tc1 cells can be differentiated under IL-12 conditions and produce IFNγ through activation of the transcription factors, such as T-bet and STAT4. Th2/Tc2 cells can be differentiated under IL-4 conditions and produce IL-4, IL-5 and IL-13 through GATA-3 and GATA-3, STAT6, respectively (48). Data have shown that Th1 and Tc1 were significantly higher, while Th2 and Tc2 were significantly lower in the peripheral blood of acute exacerbation of COPD (AECOPD) patients. This is because Th1 cells secrete IFN-γ to inhibit the proliferation of Th2 cells, which aggravates the pathological damage caused by Th1-mediated protective immunity after imbalance and promotes the pulmonary inflammatory response in COPD patients. The increase in the number of Th1 cells is also able to induce the proliferation of Tc1 cells, which in turn inhibits the proliferation of Tc2 cells (47). In peripheral small airways, Th1/Tc1 cells can secrete and release IFN-γ to act on alveolar macrophages and attract the infiltration of neutrophils (47, 50). Since Th2/Tc2 cells secrete an abundance of type II cytokines that can promote IgE production and the recruitment of pathogenic cells, such as eosinophils, these cells impose significant influence on allergy reactions, particularly in the respiratory tract (49, 51).

Th17 cells are characterized by the release of proinflammatory cytokines, such as IL-17A, IL-17F, IL-21 and IL-22, which are associated with COPD progression and the exacerbation of alveolar destruction (52, 53). They are found mainly in the bronchial mucosa and express the transcription factor RORγt as a specific marker (54). The IL-17A and IL-17F cytokines are predominantly released by Th17 cells, which are linked to neutrophilic inflammation, as IL-17 induces airway epithelial cells to release neutrophil chemotactic chemokines such as CXC-chemokine ligand 1 (CXCL1) and CXCL8, attracting neutrophils into the airways (55). In contrast, Treg cells are responsible for the regulation of immune responses by suppressing inflammation and autoimmunity through the release of anti-inflammatory cytokines, such as IL-10 and transforming growth factor-β (TGF-β) (46). Intracellular expression of Foxp3 is currently considered as the most specific marker for Treg cells and these suppressive functions are dependent on the expression of the transcription factor Foxp3 (56). In the absence of IL-10, the IL-23 levels may increase, leading to their differentiation into Th17 cells (52, 57).

Studies over the past decade has emphasized the importance of maintaining a balance between Th17 cells and Treg cells in controlling the inflammatory response in COPD (58). An increased Th17 response and Th17/Treg ratio play critical roles in the progression of COPD (52, 59). Wang et al. (60) showed that patients with moderate and severe COPD had a higher frequency of Th17 cells, elevated levels of RORγt mRNA expression and increased serum levels of IL-17A, IL-6, IL-21, IL-22 and IL-23. The authors also showed a lower frequency of Treg cells, and decreased Foxp3 mRNA expression and serum level of IL-10 in those patients. Additionally, it was demonstrated that the increase in the Th17/Treg ratio was negatively correlated with the worsening of lung function in those patients. In accordance, flow cytometric analysis revealed a significantly higher Th17/Treg ratio in the COPD group compared to non-smoking patients (59).

T-lymphocyte-mediated dysfunction of cellular immunity may have an impact in the course of AECOPD, participating in and contributing to its deterioration (47). Blood immunophenotypic analysis showed that COPD patients with low circulating lymphocyte counts (especially reduced CD4+ T-cells) were more likely to experience acute exacerbations, including persistent exacerbation (61). Similarly, Freeman et al. (62) showed that both CD4+ and CD8+ T cells were significantly reduced in AECOPD, which may indicate extravasation of T cells to sites of inflammation or organized lymphoid tissue, and then increased to a constant level at steady state. They also concluded that a decrease in CD4+ and CD8+ T cells frequency could be a potential marker of AECOPD as it precedes the onset of symptoms. However, larger studies are needed to verify this possibility.

#### 3.5.2 B lymphocytes

In the lungs of COPD patients, the number of B-lymphocytes is increased, especially in patients with severe disease (4). B lymphocytes may secrete autoantibodies against oxidized extracellular matrix proteins or against endothelial cells (46). Proteins that have been modified by carbonyl groups due to oxidative stress can stimulate the production of auto-antibodies (63). Serum anti-carbonyl-modified auto-protein antibody titers were significantly increased in patients with severe COPD compared to controls. Antibody levels were negatively correlated with disease severity and were predominantly of the IgG1 type (63). B-lymphocyte infiltration into the submucosal layer of the small airways of COPD patients has been reported, and lymphoid aggregates containing well-defined follicles and germinal centers increase with the severity of COPD disease (8, 64). In patients with very severe COPD, an increased number of B cells was found only in the connective tissue of the patients’ small airways compared to controls (65). Lymphoid follicles in the small airways of COPD patients consist of a large number of B-lymphocytes scattered with CD21+ and CD35+ follicular dendritic cells (66). B-cell activating factor plays a crucial role in regulating the function and proliferation of B-cells, and its levels are elevated in the lymphoid follicles of individuals with COPD (67). These changes may be related to the inflammatory response and disease progression in COPD.

### 3.6 Dendritic cells

Dendritic Cells (DCs) also play an important role in the pathogenesis of COPD. DCs are an important component of the immune system, mainly involved in initiating the immune response and regulating the innate immune response (68). In patients with COPD, the number of DCs in the airways is altered, and smoking causes DCs to release inflammatory chemokines, such as CCL20, which play an important role in the pathogenesis of COPD (69). Studies have shown that dendritic cell accumulation exists in the bronchial mucosa of patients who smoke and COPD, and that cigarette smoke alters dendritic cell maturation and function, thereby affecting the immune response in the lungs (70). Specifically, cigarette smoke activates epithelial cells and macrophages, causing them to release a variety of chemokines that attract and activate inflammatory cells in the lungs, including DCs (69, 71). These inflammatory cells, along with macrophages, polymorphonuclear leukocytes (PMN), and epithelial cells, release proteases such as MMP and NE, leading to elastin degradation and emphysema formation (69, 72). In addition, DCs, by releasing pro-inflammatory chemokines, may be involved in maintaining neutrophilic airway inflammation, which in turn leads to emphysematous lung tissue destruction (69). DCs are not only involved in the triggering of COPD pathogenesis, but may also play an important role in the pathologic process of COPD by modulating effector CD8+ T cell responses (69). Interventions targeting the function of DCs may provide new avenues for the treatment of COPD.

## 4 Oxidative stress

Oxidative stress is strongly associated with COPD. Smoking or dust irritation leads to lung cell damage, excessive mucus secretion and neutrophil accumulation generating large amounts of ROS, which oxidatively inactivate antiproteinases and destroy lung tissue structure. The accumulation of neutrophils also activates a large number of inflammatory factors, generating more ROS and exacerbating oxidative stress, especially during exacerbations (73, 74). Increased oxidative stress in the lungs of COPD patients has been demonstrated by measuring various markers of oxidative stress in breath. For example, ethane, a volatile product of lipid peroxidation, is increased in the exhaled breath of COPD patients and correlates with disease severity (75). COPD patients also had increased concentrations of H_2_O_2_, malondialdehyde, 4HNE (4-hydroxy-2-nonenal), and 8-isoprostane in exhaled breath condensate (76–79), and these markers increased further during exacerbations (80, 81). These increased markers of oxidative stress remained elevated even in ex-smokers, suggesting that they derive from endogenous oxidative stress and may be a reflection of ongoing lung inflammation (79). The transcription factors FOXO3a (Forkhead box O3a) and Nrf2 (nuclear factor erythroid 2-related factor 2) regulate multiple antioxidant genes, both of which are reduced in COPD lungs (82, 83).

There is growing evidence of mitochondrial dysfunction in COPD (84). This is manifested by increased mitochondrial mass, fusion, and leaky mitochondrial membranes, phenomena that result from an impaired autophagic mechanism that scavenges damaged mitochondria (i.e., mitochondrial autophagy) (85). Abnormally functioning leaky mitochondria may be a major source of ROS in COPD (86, 87).

A number of drugs have been developed that can selectively target mitochondria, but no clinical studies have been reported in patients with COPD. Given that mitochondrial dysfunction may be a major source of mitochondrial ROS in COPD, mitochondria-targeted antioxidants may have great therapeutic potential (88).

## 5 Systemic inflammation

Patients with COPD, especially during acute exacerbations, are often complicated by systemic inflammation, which often manifests itself in elevated levels of cytokines, chemokines, and inflammatory proteins or abnormal cell counts (89, 90). In addition to COPD patients, systemic inflammation can be found in patients who smoke, as evidenced by elevated systemic inflammatory markers such as C-reactive protein (CRP), interleukin-6 (IL-6), tumor necrosis factor-alpha (TNF-alpha), activated leukocytes, and fibrinogen; however, the degree of systemic inflammation is more pronounced in COPD patients (4, 91, 92). In any case, systemic inflammation in patients with COPD may contribute to their systemic manifestations and may exacerbate comorbid diseases (4). The results of a large population-based study showed that an increase in systemic inflammatory markers was associated with an increased risk of developing diabetes, cardiovascular disease, and lung cancer (93). Patients with persistent systemic inflammation have a higher mortality rate and more frequent episodes of acute exacerbation (4).

Several studies have identified the presence of potentially pathogenic microorganisms (PPM) in the lower respiratory tract of patients with stable COPD, which may lead to systemic inflammation and increased serum C-reactive protein (CRP), interleukin-8 (IL-8) levels and plasma fibrinogen (FIB) (26, 94, 95). Long-term antibiotic therapy reduces the number of acute exacerbations of COPD and improves health-related quality of life, but it also increases the risk of bacterial resistance (96). Short-term antibiotic therapy reduces the risk of bacterial resistance and drug-related side effects, but its effectiveness is inconsistent. Some studies have shown that short-term oral antibiotics can reduce airway bacterial load, decrease airway inflammation, and even eliminate PPM for a short period of time, but the bacteria rapidly recolonize. However, most studies have found that short-term antibiotic therapy does not reduce airway inflammation or decrease acute exacerbations of COPD, and may even lead to increased bacterial resistance (97). In conclusion, the outlook for the treatment of systemic inflammation remains challenging and further research is needed to clarify the efficacy of antibiotic therapy and to explore new therapeutic approaches to reduce systemic inflammation and improve patients’ quality of life.

In addition to antibiotics, statin is an important intervention for addressing systemic inflammation. Simvastatin does not prevent exacerbations in patients with COPD who do not have metabolic or cardiovascular indications for statin therapy, according to a large, multicenter, randomized trial (98). However, in an observational study of patients with COPD treated with statins for cardiovascular and metabolic indications, an association between statin use and improved outcomes, including exacerbations and reduced mortality, was reported (99). In addition, a cohort study including 950 COPD outpatients found no association between statin use and risk of AECOPD or all-cause mortality (100). This result adds to the evidence suggesting that statin therapy should not be initiated at the outset in COPD solely for prevention, but should be prescribed in line with current cardiovascular disease guidelines, particularly to reduce mortality.

## 6 Conclusion

The inflammation pathogenesis of COPD is complex, mainly related to inflammatory cell, oxidative stress, and systemic inflammation. COPD encompasses several different clinical and pathophysiologic phenotypes, and it is essential to identify COPD phenotypes that are efficacious for specific therapies, which includes identifying disease types and biomarker testing. On the basis of the foregoing, AAT augmentation therapy and mitochondria-targeted antioxidants may have great therapeutic potential. Corticosteroids have shown more significant efficacy in COPD patients with eosinophilic inflammation. However, roflumilast, a phosphodiesterase-4 inhibitor, appears to be more effective in patients with a neutrophilic phenotype, which may become a therapeutic agent for the neutrophil-mediated inflammatory response (1, 101, 102). In patients prone to acute exacerbations, studies have shown that regular use of certain antibiotics such as azithromycin or erythromycin for one year reduces the risk of acute exacerbations (103). In addition to antibiotics, statin is an important intervention for addressing systemic inflammation in COPD.

Therefore, there is a greater need to use specific biomarkers to predict the effect of treatment, such as specific proteins like serine proteases. While much of the focus and difficulty is on patients with advanced disease, numerous studies have shown that patients in the early stages of the disease have a greater therapeutic benefit, and if effective, precise and safe anti-inflammatory therapies can be developed for different phenotypes of COPD, intervening in the early stages of the disease will show greater benefit, preventing disease progression and reducing the burden of disease.
